# Multimodal magnetic resonance imaging investigation of basal forebrain damage and cognitive deficits in Parkinson's disease

**DOI:** 10.1002/mds.27561

**Published:** 2018-12-10

**Authors:** Fatma Gargouri, Cécile Gallea, Marie Mongin, Nadya Pyatigorskaya, Romain Valabregue, Claire Ewenczyk, Marie Sarazin, Lydia Yahia‐Cherif, Marie Vidailhet, Stéphane Lehéricy

**Affiliations:** ^1^ Brain and Spine Institute – ICM Center for NeuroImaging Research – CENIR Paris France; ^2^ Sorbonne Université, University Pierre and Marie Curie Paris 06 Inserm U1127, Centre National de la Recherche Scientifique (CNRS) Unité Mixte de Recherche (UMR) 7225 Paris France; ^3^ Brain and Spine Institute (ICM) Team Movement Investigation and Therapeutics Paris France; ^4^ Department of Neurology Pitié‐Salpêtrière Hospital, Assistance Publique Hôpitaux de Paris (APHP) Paris France; ^5^ Department of Neuroradiology Pitié‐Salpêtrière Hospital, Assistance Publique Hôpitaux de Paris (APHP) Paris France; ^6^ Department of Neurology, Memory and Language Paris Descartes University, Sorbonne Paris Cité, Sainte Anne Hospital Paris France; ^7^ Research unit 1023 Inserm/Commissariat à l'Energie Atomique (CEA)/University Paris In vivo Molecular Imaging Laboratory (IMIV)

**Keywords:** cognition, cholinergic, acetylcholine, diffusion MRI, functional connectivity, resting state fMRI

## Abstract

**Background:**

Cognitive deficits in Parkinson's disease (PD) may result from damage in the cortex as well as in the dopaminergic, noradrenergic, and cholinergic inputs to the cortex. Cholinergic inputs to the cortex mainly originate from the basal forebrain and are clustered in several regions, called Ch1 to Ch4, that project to the hippocampus (Ch1‐2), the olfactory bulb (Ch3), and the cortex and amygdala (Ch4).

**Objective:**

We investigated changes in basal forebrain and their role in cognitive deficits in PD.

**Methods:**

We studied 52 nondemented patients with PD (Hoehn & Yahr 1‐2) and 25 age‐matched healthy controls using diffusion and resting state functional MRI.

**Results:**

PD patients had a loss of structural integrity within the Ch1‐2 and Ch3‐4 nuclei of the basal forebrain as well as in the fornix. Tractography showed that the probability of anatomical connection was decreased in PD between Ch3‐4 and the associative prefrontal cortex, occipital cortex, and peri‐insular regions. There was a reduction in functional connectivity between Ch1‐2 and the bilateral hippocampi and parahippocampal gyri, the left middle and superior temporal gyri, and the left fusiform gyrus and between Ch3‐4 and the right inferior frontal gyrus and the right and left thalamus. In Ch1‐2, loss of structural integrity and connectivity correlated with scores at the memory tests, whereas changes in Ch3‐4 correlated with scores of global cognition and executive functions.

**Conclusion:**

This study highlights the association between deficits of different cholinergic nuclei of the basal forebrain and the extent of cognitive impairments in nondemented PD patients. © 2018 The Authors. *Movement Disorders* published by Wiley Periodicals, Inc. on behalf of International Parkinson and Movement Disorder Society.

Cognitive deficits are frequently observed in patients with Parkinson's disease (PD). After 10 years, approximately 75% of PD patients develop dementia.^1^, ^2^, ^3^ The origin of cognitive dysfunction in PD is multifactorial in relation to damage of the cortex and of several neurotransmission networks, including dopaminergic, noradrenergic, and cholinergic systems.^4^ A dual syndrome model has been proposed to explain these cognitive deficits,^5^ which postulate that dopaminergic deficits may result in fronto‐striatal executive dysfunction, whereas dementia may be a consequence of damage to other systems, including cholinergic and posterior cortical structures.^6^, ^7^, ^8^, ^9^, ^10^, ^11^, ^12^

There are 2 main types of cholinergic projection neurons located in the pedunculopontine nucleus, which innervates the thalamus, striatum, cerebellum, and brain stem, and in the basal forebrain, which innervates the cortex, amygdala, and hippocampus. The basal forebrain is divided into different nuclei including the medial septal nucleus (Ch1), the nucleus of the vertical limb of the diagonal band (Ch2), the nucleus of the horizontal limb of the diagonal band (Ch3), and the nucleus basalis of Meynert (Ch4). These nuclei have distinct connections. Ch1 and Ch2 project to the hippocampus, Ch3 to the olfactory bulb, and Ch4 to associative cortical regions and the amygdala.^13^ Ch4 is further divided in smaller nuclei with different connections.^13^ The basal forebrain is difficult to delineate using MRI because it does not present precise anatomical borders. Several approaches have been proposed including simple thickness measurements of the substantia innominata on coronal T2‐weighted images^14^ or automated measurements using a mask of the structure derived from combined histology and postmortem brain MRI.^15^

In PD, histological studies have reported neuronal loss in the nucleus basalis of Meynert as well as the presence of Lewy bodies and α‐synuclein in this structure.^16^, ^17^ Cell loss in the nucleus basalis of Meynert and α‐synuclein aggregation were greater in PD patients with dementia when compared with nondemented PD.^17^ Although no cell loss was observed in Ch1‐2, Lewy bodies pathology was present as well as reduced choline acetyltransferase activity in the output structure, the hippocampus.^17^ Cortical cholinergic deficit was observed using positron emission tomography (PET) tracer of acetylcholinesterase activity in PD with and without dementia^18^, ^19^, ^20^ with increasing frequency of cortical cholinergic denervation in patients with greater cognitive impairment.^21^ Using MRI, decreased volume of the substantia innominata was reported in PD patients with normal or altered cognition.^22^, ^23^ Decreased volume and increased mean diffusivity in the nucleus basalis was also predictive of cognitive decline in cognitively intact PD.^24^, ^25^ The cognitive deficits in PD that may relate to cholinergic dysfunction affect visuospatial learning and memory functions in tasks such as visual pattern and spatial recognition memory, visuospatial paired associative learning, and visual simultaneous and delayed‐matching‐to sample tasks^6^, ^26^, ^27^, ^28^ as well as tests of attention and executive function,^22^, ^29^ particularly in processes underlying the immediate registration of information.^30^

In this study, we characterized damage of the basal forebrain in PD using multimodal MRI and further investigated the relationships between basal forebrain changes and cognitive deficits in nondemented PD patients. We first used diffusion MRI (dMRI) to quantify structural changes in the different nuclei of the basal forebrain from Ch1 to Ch4. Second, we used diffusion‐based tractography and resting state functional MRI (rsfMRI) to quantify changes in anatomical and functional connectivity respectively between Ch1‐2 and the hippocampus via the fornix and between Ch3‐4 and the cortex. Third, we studied the relationships between cognitive evaluations and (1) structural changes in the basal forebrain and (2) changes in anatomical and functional connectivity abnormalities between the basal forebrain nuclei and their respective cortical connections.

## Material and Methods

### Participants

Clinical characteristics of the participants are presented in Table 1. A total of 52 PD patients with mild to moderate severity and 25 healthy volunteers (HV) were prospectively recruited in the Movement Disorders clinic of the Pitie‐Salpetriere Hospital between April 2010 and September 2012 (Nucleipark project). The patients had to meet the following inclusion criteria: clinical diagnosis of idiopathic PD made by a movement disorder specialist according to Queen Square Brain Bank Criteria,^31^ age between 18 and 75 years, no or minimal cognitive disturbances (Mini‐Mental State Examination >24). PD had mild cognitive impairment (MCI) if they fulfilled the criteria of the MDS Task Force on MCI in PD.^2^ The exclusion criteria were any additional neurological disorder. Normal HV had no history of neurological disorders. HV with mild cognitive impairment were excluded. Patients and HV were also excluded if there were contraindications to MRI, abnormalities on the MRI scans, or psychiatric disorders. All participants gave their written informed consent. The study was approved by the local ethics committee (Comité de Protection des Personnes (CPP) Paris VI, Registration number of the study (ID‐RCB): 2009‐A00922‐55).

**Table 1:** Demographical, clinical, and neuropsychological data

Data		HV	PD	*P* value
**Demographic data**				
Number of subjects		25	52	
Males/females		13/12	37/15	.05^a^
Age, y		59.8 ± 8.0	60.6 ± 8.8	.71
Disease duration		–	8.7 ± 3.5	
Education		5.68 ± 1.49	5.19 ± 1.55	.19
**Motor clinical data**				
Hoehn and Yahr stage, 0–5		–	1.88 ± 0.63	
UPDRS III (“best‐on” condition), 0–108		0.6 ± 0.1	16.4 ± 8.8	**<.001**
Levodopa equivalent daily dose (mg)		–	786.7 ± 290.62	
**Neuropsychological tests**				
Mattis Dementia Rating Scale, 0–144		140.0 ± 2.6	136.0 ± 9.4	.12
Mini‐Mental State Evaluation, 0–30		28.7 ± 1.1	27.8 ± 1.8	**.02**
Montgomery Asberg Depression Rating Scale		4.32 ± 4.25	9.00 ± 5.47	**<.001**
Epworth scale		4.52 ± 3.00	8.73 ± 5.07	**<.001**
**Free and Cued Selective Reminding Test**				
Immediate free recall/16		15.6 ± 0.7	14.5 ± 1.7	**<.001**
Total free recall/48		33.1 ± 4.9	28.2 ± 6.8	**<.01**
Total recall/48		47.2 ± 1.4	45.2 ± 3.1	**<.01**
Delayed free recall/16		12.5 ± 2.1	11.1 ± 3.2	**.03**
Delayed total recall/16		15.8 ± 0.6	15.4 ± 1.1	.05
**WAIS III**				
Code		67.8 ± 13.7	51.6 ± 13.5	**<.001**
Letter number		9.8 ± 3.4	8.5 ± 3.0	.07
Trail‐Making Test B‐A		43.3 ± 32.6	55.9 ± 35.4	.12
**Stroop**				
Denomination		76.2 ± 15.0	66.4 ± 10.6	**<.01**
Reading		99.5 ± 14.5	92.1 ± 14.7	**.04**
Interference		40.0 ± 6.1	35.5 ± 9.8	**.03**
**Rey figure**				
Recall		34.9 ± 1.3	34.1 ± 2.5	.14
Copy		18.4 ± 4.5	19.7 ± 4.9	.63

Significant *P* values are indicated in bold. Chi2, chi‐squared test; HV, healthy volunteer; UPDRS, Unified Parkinson's Disease Rating Scale; WAIS, Wechsler Adult Intelligence Scale.

a Groups were compared using *t*‐tests except Chi2.

### Clinical and Neuropsychological Evaluation

The tests were performed in the “best‐ON” condition and are presented in Table 1. Motor impairment was assessed using the UPDRS III. The neuropsychological assessment included the Mini‐Mental State Examination (MMSE) and Mattis Dementia Rating Scale for global cognitive efficiency, Trail Making Test (TMT) and the Stroop test for executive functions, and the Free and Cued Selective Reminding Test for verbal episodic memory, copy, and recall of Rey's figure for visuoconstructive function and visual memory. Depression was evaluated with the Montgomery and Asberg Depression Rating Scale (MADRS). Sleepiness was evaluated with the Epworth scale.

### Neuroimaging

#### *Data Acquisition*

Images were acquired using a 3 T Siemens TRIO 32‐channel TIM system (Siemens, Erlangen, Germany) with a standard 12‐channel head coil for signal reception. The MRI protocol included the following acquisitions: sagittal 3‐dimensional T1‐weighted images (repetition time = 2200 milliseconds, echo time = 2.9 milliseconds, flip angle = 10°, 1 mm isovoxel size); 3‐dimensional T2‐weighted images (repetition time = 3000 milliseconds, echo time = 300 milliseconds, flip angle = 120°, 1 mm isovoxel size); dMRI (48 axial spin echo echo planar slices, 60 directions with a b‐value of 1000 s/mm^2^ and 3 b0 images; 1.7 mm isovoxel size, antero‐posterior phase‐encoding direction), blood oxygen level dependent echo planar images for rsfMRI (axial plane, repetition time = 2900 milliseconds, echo time = 30 milliseconds; flip angle = 90°, 200 volumes; 3 mm isovoxel size, antero‐posterior phase‐encoding direction). During the rsfMRI acquisition, the participants kept their eyes closed and had to relax and remain awake. MRI was performed in the OFF condition (treatment withdrawal for 12 hours before the beginning of the acquisition).

#### *Regions of Interest*

##### *Seed Regions: Segmentation of Ch1‐2 and Ch3‐4*

MRI segmentation of regions Ch1 to Ch4 was based on histological sections that were obtained in the frame of previous histological study (Supplementary Fig. 1).^32^ The histological procedure is described in the supplementary material.^32^

**Figure 1:**
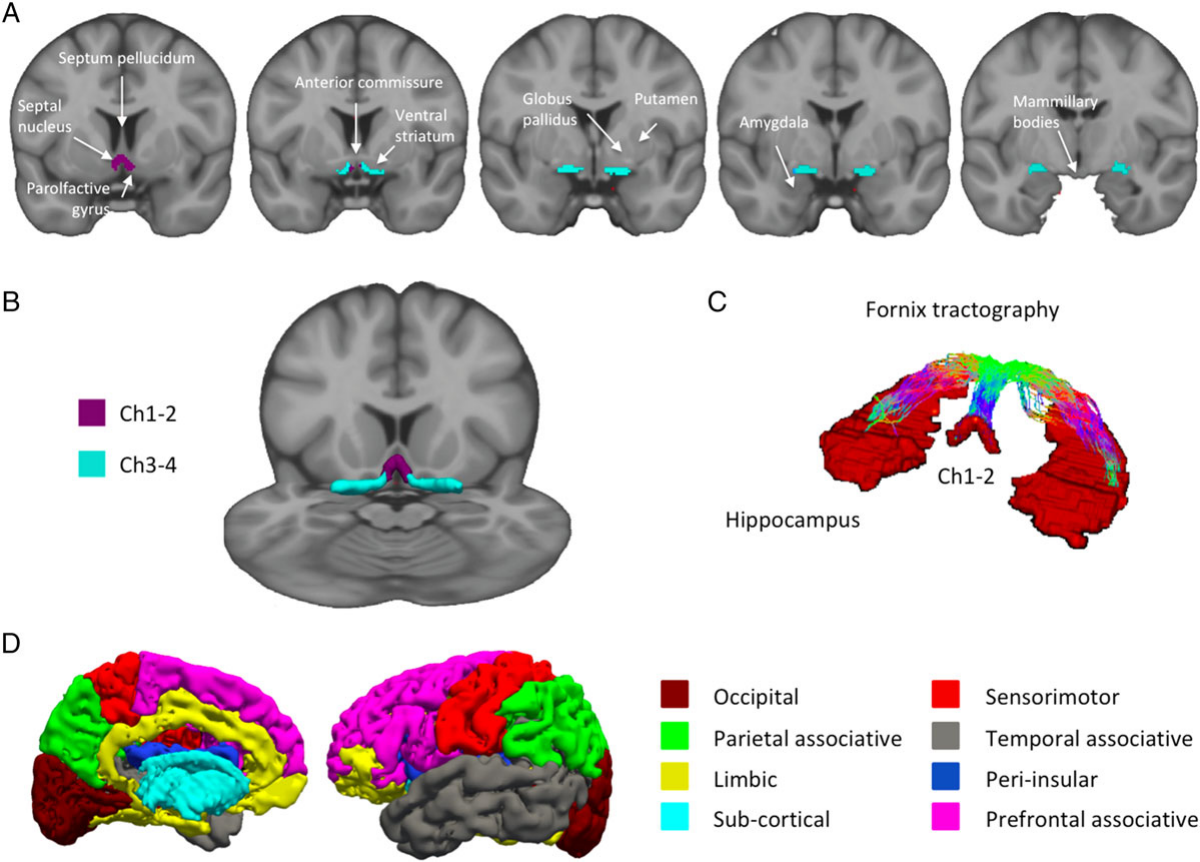
MRI‐based segmentation of Ch1‐Ch4. (A) Coronal view of Ch1‐2 (purple) and Ch3‐4 (light blue) regions of interest superimposed on coronal T1‐weighted Montreal Neurological Institute template image. (B) Three‐dimensional representation of the regions of interest on a multiplanar view of the Montreal Neurological Institute template, (C) Fornix tractography between Ch1‐2 and the hippocampi, and (D) Freesurfer segmentation of the cortical and subcortical areas.

Using MRI, normalized masks of Ch1‐2 and Ch3‐4 were created by manual segmentation independently by 2 examiners in 10 HV using coronal 3‐dimensional T1‐weighted and T2‐weighted images (Fig. 1). Each examiner performed MRI segmentation of Ch1 to Ch4 regions in the coronal plane in the native space by comparison with histological sections in each of these 10 participants. Ch1 and Ch2 were segmented together because they were difficult to delineate one from another and they both project to the hippocampal complex.^13^ Similarly, Ch3 and Ch4 were segmented together as they were difficult to delineate one from another. The borders of Ch1‐2 were defined as follows: the anterior part of Ch1‐2 corresponded to the lateral septal nucleus, dorsal to the parolfactive gyrus, ventral to the septum pellucidum, and medial to the striatum. At this level, the ventral striatum connected the caudate and putamen. The posterior limit of Ch1‐2 was arbitrarily defined as the slice including the rostral border of the anterior commissure. Ch3‐4 was located caudal to this level, ventral to the ventral striatum (rostrally) and the putamen/globus pallidus (caudally), and dorsal to the amygdala. The posterior limit of Ch3‐4 was arbitrarily defined as the last slice containing the mammillary body and usually the first slice containing the hippocampus. The segmentation results are presented in Figure 1. Dice and Jaccard scores between the 2 segmentations performed by the 2 examiners were then calculated (interrater variability). The values of the Dice and Jaccard scores were calculated for both Ch1‐2 (Dice = 0.64; Jaccard = 0.47) and Ch3‐4 (Dice = 0.79; Jaccard = 0.64) and were significant (*P* < .001), indicating good interindividual reproducibility of segmentations.

We then created a mean mask per participant corresponding to the intersection of the 2 masks of both tracers resulting in 1 mean mask in the native space for each of the 10 participants. Each mean mask was normalized to the Montreal Neurological Institute (MNI) space by using the normalization function of SPM software (Statistical Parametric Mapping, https://www.fil.ion.ucl.ac.uk/spm/), resulting in 10 mean masks in the MNI space. Last, we created a group mean mask corresponding to the intersection of all mean masks of the 10 participants. The resulting group mean Ch12 and Ch34 masks were used for the diffusion and fMRI analyses.

##### *Target Regions: Segmentation of the Hippocampus and Cortical and Subcortical Regions*

For the resting state fMRI analysis, we defined target regions on the basis of the known anatomical connections of the basal forebrain, that is, the hippocampus for Ch1 and Ch2 and the cortex for Ch4.^13^ We used the mask of the hippocampus extracted from the Automated Anatomic Labeling in the MNI space because all analyses were performed on the MNI space. For the diffusion analysis, we defined the hippocampus and the cortical regions from the Freesurfer parcellation^33^ because we performed the analysis on the native space. We defined the following 8 cortical regions: the associative parietal, associative prefrontal, associative temporal, limbic, occipital, sensorimotor and peri‐insular cortex, and subcortical nuclei (Fig. 1, Supplementary Table 1).

#### *Diffusion MRI Analysis*

Data preprocessing for dMRI was performed by using standard pipeline (distortion correction with field MAP and motion and eddy‐current with EDDY from FSL software; www.fsl.fmrib.ox.ac.uk/fsl/fslwiki/eddy). The tensor metrics included fractional anisotropy (FA), mean diffusivity (MD), axial diffusivity (AD), and radial diffusivity (RD) and were computed by using dtifit tools from FSL. The dMRI analysis was performed in the native space. Thus we denormalized the group mean Ch1‐2 and Ch3‐4 masks to each individual's native space by using SPM. Within the denormalized Ch1‐2 and Ch3‐4 masks, we calculated the mean of each diffusion metric (FA, MD, AD, and RD) by averaging individual values of each voxels contained within the masks.

We then studied basal forebrain structural connectivity using tractography. For Ch1‐2 connections, we defined the fornix in each participant space. We performed probabilistic tractography based on the constrained spherical deconvolution with mrtrix software (http://www.mrtrix.org/). We generated the fornix tracks by seeding from Ch1‐2 to hippocampus (target). We converted these tracks into a density map and kept voxels containing more than two tracks to define the fornix mask. We further excluded voxels belonging to the CSF by using the CSF map obtained by segmenting the anatomical scan with spm_segment. There was no between‐group difference in the volumes of the fornix masks (HV = 1519 ± 623 mm^3^, PD = 1324 ± 709 mm^3^, *t*‐test *P* = .26). To reconstruct the tracts, we used exclusion masks that corresponded to the right hemisphere for tracks in the left hemisphere (eg, exclusion mask of the right hemisphere to reconstruct tracts between the left Ch1‐2 and the left hippocampus and tracts between the left Ch3‐4 and the left cortical areas) and vice versa.

For tractography‐based connectivity, we used bedpostx and probtrackx tools, with standard options to create the density of probability carts between Ch1‐2 and the hippocampus and between Ch3‐4 and the 7 cortical masks and the subcortical mask. We intersected the track density map with each cortical mask and summed the number of fibers within the mask. We used the same statistical model to compare HV and PD for the density of probability.

#### *Resting‐State fMRI Analysis*

Data preprocessing for fMRI was performed by using the toolboxes DPARSF^34^ and fMRI Data Analysis Toolkit REST,^35^ which were based on Statistical Parametric Mapping (SPM8). Preprocessing included slice timing: the functional images were interpolated in time to correct phase advance during volume acquisition and realigned to the first image of each session. Nuisance covariates were regressed out individually (head movements, white matter, and cerebrospinal fluid signals). To evaluate head movements, we used the 6 translations/rotations parameters. We calculated the frame displacement as defined in ref. 36. There were no significant differences in frame displacement between the 2 groups (mean ± standard deviation, HV = 0.25 ± 0.12; PD = 0.22 ± 0.11; analysis of variance *P* = .32, F = 1.01).

The structural T1‐weighted scans were normalized using DARTEL and the MNI152 NLIN sixth generation template of SPM8. The fMRI scans were normalized by applying DARTEL registration parameters of the coregistered structural MRIs by using the DPARSF tool box in SPM8 and smoothed with a 6‐mm Gaussian filter. Then, linear detrending and temporal bandpass (0.01–0.08 Hz) filtering were performed to remove low‐frequency drifts and physiological high‐frequency noise.^37^

Normalized masks of Ch1‐2 and Ch3‐4 were taken as seed ROIs to test our hypotheses. Time series were extracted from the seed ROIs and entered as regressors in a global linear model in SPM8 (first‐level analysis). Then we defined the second level by comparing group effect between HV and PD.

### Statistical Analysis

A 1‐way analysis of covariance was conducted to compare diffusion variables (FA, MD, AD, and RD) inside the fornix, Ch1‐2 and Ch3‐4, between the groups (HV, PD) while controlling for age, gender, education, scores at the MADRS and Epworth scales, and the levodopa equivalent daily dose. Pearson correlations between diffusion variables and clinical scores that differed between patients and controls were computed in the patient group only controlling for age, gender, education, scores at the MADRS and Epworth scales, and the levodopa equivalent daily dose. All *P* values were adjusted for multiple comparisons using the false discovery rate method for analysis of covariance^38^ and permutation test for Pearson correlations.^39^

For rsfMRI functional connectivity analysis, we ran a 1‐way analysis of variance with 2 levels (PD, HV) to evaluate the difference between the groups. Then we ran a regression analysis in the patient group to look for possible correlations between Ch1‐2–hippocampus connectivity, Ch3‐4–cortex connectivity, and the neuropsychological measures that showed specific effects in the PD versus control comparisons. We applied a mask of the brain without the cerebellum. Activation maps were first thresholded at *P* < .001 uncorrected. In these maps, clusters were considered significant at *P* < .05 corrected for multiple comparisons using family wise error unless stated otherwise. All fMRI statistics were performed by using age, gender, education, scores at the MADRS and Epworth scales and levodopa equivalent daily dose as covariates.

## Results

### Patients

Patients were matched for age and education. There were more male and female PD patients, but the difference in ratios between the groups did not reach significance. No patient had anticholinergic medication. Of the 52 PD patients, 20 fulfilled the criteria for MCI. Patients differed from controls for scores at the MMSE, MADRS, Epworth, Free and Cued Selective Reminding Test, Wechsler Adult Intelligence Scale (WAIS) III code, and Stroop test. Scores did not differ for Mattis Dementia Rating Scale, WAIS III Letter Number, TMT B‐A, and copy and recall of Rey figure. As imaging measurements did not differ between MCI and non‐MCI patients, they were analyzed together.

### Diffusion Data Analysis

In PD patients, MD, AD, and RD increased in Ch1‐2; MD and RD increased in Ch3‐4; and there was a trend for AD in Ch3‐4 (Table 2). In the fornix, only AD was increased, and there was a trend for MD (Table 2).

**Table 2:** Diffusion changes in the basal forebrain and fornix

Region	Diffusion measure	Healthy volunteers	PD patients	ANCOVA *P* value
Fornix	FA	0.213 ± 0.036	0.212 ± 0.037	.527
	MD	0.961 ± 0.086	1.025 ± 0.109	.077
	AD	1.151 ± 0.089	1.214 ± 0.105	**.040**
	RD	0.870 ± 0.085	0.924 ± 0.113	.151
Ch1‐2	FA	0.171 ± 0.022	0.168 ± 0.021	.876
	MD	0.796 ± 0.080	0.853 ± 0.081	**.029**
	AD	0.915 ± 0.091	0.977 ± 0.080	**.029**
	RD	0.737 ± 0.076	0.790 ± 0.081	**.029**
Ch3‐4	FA	0.210 ± 0.026	0.202 ± 0.025	367
	MD	0.744 ± 0.089	0.805 ± 0.082	**.034**
	AD	0.875 ± 0.103	0.948 ± 0.083	.053
	RD	0.678 ± 0.083	0.735 ± 0.084	**.034**

Values are in mm.s^−2^ × 10^−3^. Significant differences are indicated in bold. AD, axial diffusivity; ANCOVA, analysis of covariance; FA, fractional anisotropy; MD, mean diffusivity; RD, radial diffusivity.

### Connectivity Analysis

#### *Structural Connectivity*

Probtrack streamlines from Ch1‐2 reached the hippocampus through the fornix (Fig. 1). Streamlines from Ch3‐4 reached the cortex and are presented in Supplementary Figure 2. Ch1‐2 showed no significant changes in the probability of connection density with the hippocampus. Ch3‐4 showed significant decrease in the probability of connection density with the associative prefrontal cortex, occipital cortex, and peri‐insular regions (Supplementary Table 1).

#### *Functional Connectivity*

**Figure 2:**
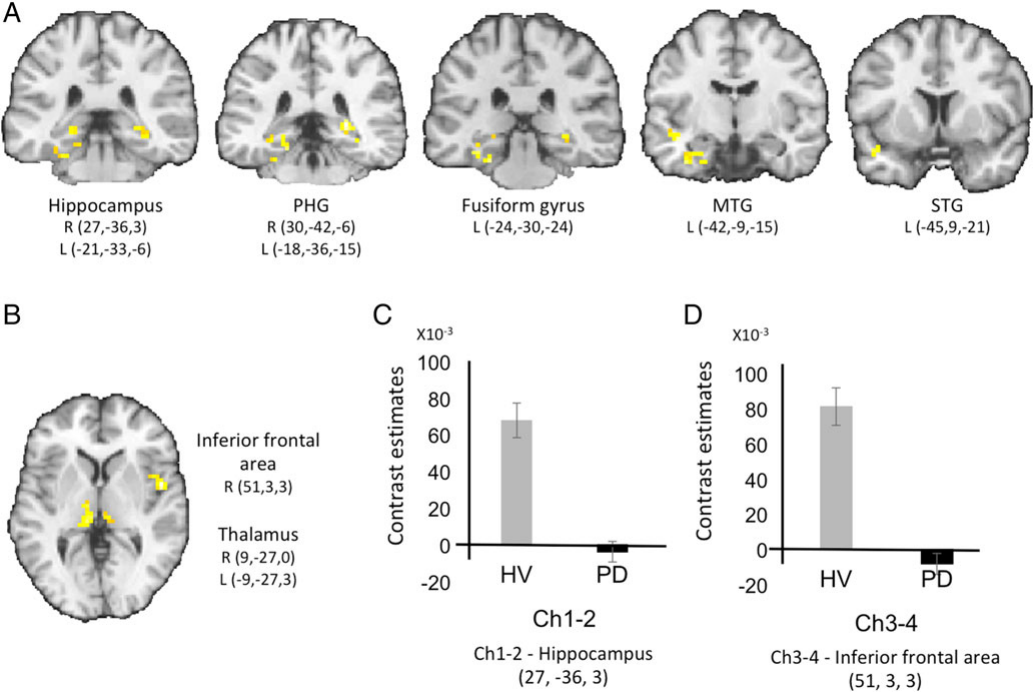
Results of the functional connectivity analysis. PD patients showed reduced functional connectivity (A) between Ch1‐2 and the hippocampus, the parahippocampal gyrus, the middle and superior temporal gyri and the fusiform gyrus, and (B) between Ch3‐4 and the inferior frontal and the thalamus. Cluster significant at *P* < .05, family wise error corrected for multiple comparisons, left is left. C shows the contrast estimates for Ch1‐2–hippocampus connectivity and D shows the contrast estimates for Ch3‐4–inferior frontal area connectivity, respectively. HV, healthy volunteers; L, left; MTG, middle temporal gyrus; STG, superior temporal gyrus; PD, Parkinson's disease; PHG, parahippocampal gyrus; R, right.

Maps of functional connectivity main effects are presented in Supplementary Figure 3. PD patients showed reduced functional connectivity between Ch1‐2 and bilateral hippocampi and parahippocampal gyri, the left fusiform gyrus, and the left superior and middle temporal gyri (Fig. 2, Supplementary Table 2). PD patients showed reduced functional connectivity between Ch3‐4 and the right inferior frontal area and bilateral thalamus (Fig. 2, Supplementary Table 2). Using a more liberal uncorrected threshold of *P* < .01 uncorrected for multiple comparisons, PD patients showed a trend for reduced functional connectivity between Ch3‐4 and the temporo‐occipital regions (Supplementary Fig. 4).

### Correlation With Cognitive Functions

**Figure 3:**
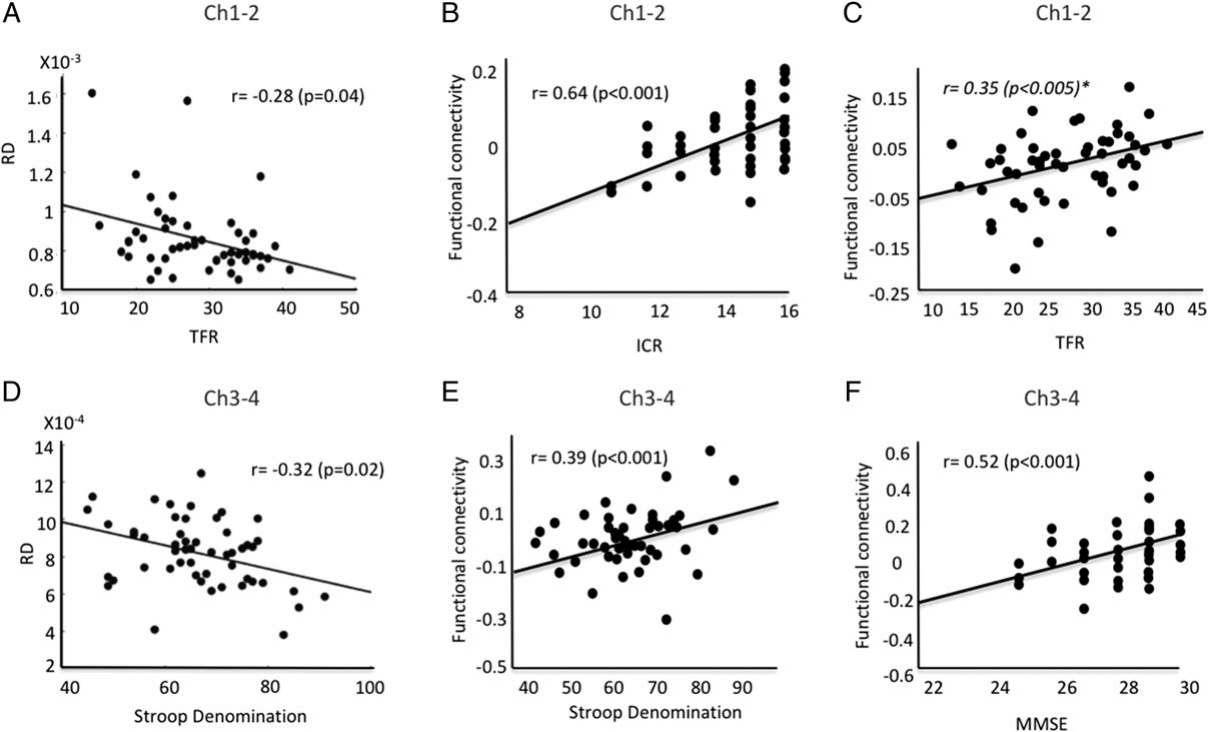
Correlations between clinical scores and imaging measures. (A) Radial diffusivity (RD) in Ch1‐2 correlated with scores at the total free recall (TFR) and (D) radial diffusivity in Ch3‐4 correlated with scores at the Stroop denomination test. (B) Functional connectivity changes in Ch1‐2 correlated with scores at the immediate cued recall (ICR) in the hippocampus. (C) There was a correlation trend for total free recall (TFR) in the superior frontal gyrus. (E) Functional connectivity changes in Ch3‐4 correlated with scores at the Stroop denomination test in the superior parietal cortex and (F) at the MMSE in the middle frontal gyrus. ICR, immediate cued recall; MMSE, mini mental status examination; RD, radial diffusivity; TFR, total free recall.

Diffusion changes in the basal forebrain in PD patients correlated with scores at several clinical tests. In Ch1‐2, there were significant negative correlations between the Total Free Recall and MD and RD (Fig. 3, Supplementary Table 3). In Ch3‐4, there were significant negative correlations between scores at the Stroop denomination and MD, AD, and RD (Fig. 3, Supplementary Table 3). In the fornix, FA correlated positively with the Total Free Recall (Supplementary Table 3). The probability of connection density between Ch1‐2 and the hippocampus did not correlate with scores at the clinical tests. The probability of connection density between Ch3‐4 and the prefrontal associative cortex correlated with scores at the MMSE (*r* = 0.42; *P* = .001; Supplementary Fig. 5). Exploratory analyses were also conducted with the other cognitive tests that showed significant negative correlations between scores at the copy of Rey Complex Figure and MD, AD, and RD in Ch1‐2 and positive correlations between TMT B‐A and RD in Ch3‐4 (Supplementary Fig. 6).

Functional connectivity changes in Ch1‐2 correlated with Immediate Cued Recall in the bilateral superior, inferior, and superomedial frontal gyri, cingulate gyrus, the left middle frontal gyrus, putamen, and hippocampus and Total Recall in the left inferior parietal lobe (Fig. 3, Supplementary Table 4 and Supplementary Fig. 7A). There was a correlation trend for Total Free Recall in the right superior frontal gyrus (*P* < .005, false discovery rate corrected; Fig. 3).

Functional connectivity changes in Ch3‐4 correlated with scores at the MMSE in the right middle frontal gyrus, bilateral superior frontal gyri, the left middle cingulate gyri, and the right thalamus, and at the Stroop denomination test in the left supplementary motor area and lateral premotor cortex, bilateral postcentral and middle cingulate gyri, and the right superior parietal cortex (Fig. 3, Supplementary Table 4, and Supplementary Fig. 7B). There were no other correlations between imaging and clinical measures. Last, there were no correlations between the movement parameters and cognitive scores.

## Discussion

PD patients presented evidence of structural changes and abnormal structural and functional connectivity in the basal forebrain. Both Ch1‐2 and Ch3‐4 showed increased MD and RD, indicating the presence of altered microstructure. Ch1‐2 presented reduced functional connectivity with the medial temporal lobe as well as abnormal diffusion measures in the fornix. In contrast, Ch3‐4 presented reduced structural and functional connectivity with associative frontal, occipital, and peri‐insular areas and medial/pulvinar thalamus. Last, changes in diffusion measures and connectivity in Ch1‐2 correlated with scores at the memory test (Free and Cued Selective Reminding Test) and the Copy of Rey's figure, whereas changes in Ch3‐4 correlated with scores of global cognition (MMSE) and executive functions (TMT B‐A and Stroop test). This study highlights the association between changes in the different cholinergic basal forebrain nuclei and the extent of cognitive impairments in PD, before the development of dementia.

Using diffusion MRI, we demonstrated in vivo that patients with mild to moderate PD presented pathological changes in both Ch1‐2 and Ch3‐4 in the basal forebrain. These results are in line with previous postmortem histological and in vivo PET and MRI studies. Several histological studies have reported severe loss of cholinergic neurons in the basal forebrain neurons in Parkinson's disease.^40^, ^41^ Based on the presence of α‐synuclein‐immunopositive Lewy neurites and Lewy bodies, Braak and colleagues^16^ have proposed that the magnocellular nuclei of the basal forebrain are affected during stage 4 of Parkinson's disease. Recently, using stereological analyses, cell loss and increase in α‐synuclein immunoreactive Lewy bodies and neurites were reported in neurons of the Ch4 nucleus basalis of Meynert in PD with dementia when compared with nondemented PD along with a reduction in choline acetyltransferase activity in the cortex.^17^ Although there was no significant cell loss in Ch1‐2 in PD patients with dementia, an increased Lewy pathology and reduced choline acetyltransferase activity in its output structure was observed in these patients.^17^ Using PET tracers of acetylcholinesterase activity, cortical cholinergic deficit was observed in the cortex as well as in the hippocampus in PD with dementia that was greater than in Alzheimer's disease.^18^, ^19^ The frequency of cortical cholinergic denervation increased with increasing cognitive impairment from 24.7% in the minimally affected group to 85.7% in the severely affected group.^18^ Higher mean diffusivity was predictive of cognitive decline in cognitively intact PD patients.^24^ Lower basal forebrain volume in the Ch3‐4 area was also reported in PD patients with or without cognitive impairment that was predictive of cognitive decline.^22^, ^23^, ^24^, ^25^ Cortical cholinergic deficit showed a frontal to occipital gradient, with larger deficits in posterior occipital regions.^20^ Consistent with this result, we found a reduction in anatomical connections between Ch3‐4 and the occipital cortex and a trend for reduced functional connections. There was also a trend for anatomical and functional connections with the temporal lobes. However, in contrast to PET studies, we did not find that occipital regions were more affected than frontal regions. This discordance may be the result of differences in the techniques used or to differences in the populations studied.

Changes in structural and functional connectivity were observed between Ch1‐2 and Ch3‐4 and several cortical and subcortical regions that corresponded with the respective projection areas of these regions. Functional connectivity maps of Ch1‐2 and Ch3‐4 were in agreement with previous studies.^42^, ^43^ In Ch1‐2, reduced functional connectivity was observed with both hippocampi, consistent with the known projections of this region,^44^ and the parahippocampal gyrus, which receives afferents from Ch2 although less than Ch4.^44^ There was no significant functional connectivity between Ch1‐2 and the cortex that receives afferents from Ch4, except for the left medial and inferior temporal lobe adjacent to the hippocampus,^44^ a finding that may be explained by indirect functional connections. Abnormal diffusion measures were observed in the fornix connecting Ch1‐2 and the hippocampus.^44^ In contrast, Ch3‐4 showed functional connectivity deficits with the frontal and thalamic areas, but not the hippocampi. The different pattern of connectivity between Ch1‐2 and Ch3‐4 is consistent with previous studies in HV that showed large cortical connections for the nucleus basalis versus more circumscribed coupling with the orbitofrontal cortex and hippocampal complex for the septal area and diagonal band of Broca.^42^, ^43^ Connectivity changes in the thalamus were unexpected as cholinergic thalamic afferents originate mostly from the pedunculopontine nucleus in the brain stem.^13^ However, the medial and pulvinar areas of the thalamus are connected to the associative cortical areas that receive afferents from Ch3‐4. Therefore, it is possible that functional connectivity between Ch3‐4 and the thalamus were indirect passing through polysynaptic pathways, as shown in nonhuman primate studies.^45^ Last, increase functional connectivity of the substantia innominata with the cortex was reported in drug‐naïve PD patients, suggesting that functional connectivity changes differ between early and more advanced stages of the disease.^46^

Structural changes were clinically relevant as they correlated with cognitive dysfunction in PD patients. Correlations were observed with performances at tests of memory, visuospatial, and executive functions as well and global cognition in line with previous behavioral,^6^, ^26^, ^27^, ^28^, ^30^ PET,^18^, ^19^, ^29^ and MRI imaging studies.^22^ Cognitive deficits in PD that were related to cholinergic dysfunction or basal forebrain structural changes were observed for visuospatial learning and memory tasks (including visual pattern and spatial recognition memory, visuospatial paired associative learning, simultaneous and visual delayed‐matching‐to sample tasks)^6^, ^25^, ^26^, ^27^, ^28^ as well as tests of attention and executive function (TMT B‐A, Stroop, WAIS‐II digit span),^22^, ^29^ particularly in processes underlying the immediate registration of information.^30^ The cognitive deficits that correlated with structural and functional changes of basal forebrain cholinergic circuits also agreed with those produced by anticholinergic drugs, which resulted in impairment in memory processes (the immediate registration of information during the Brown‐Peterson Short‐Term Memory Test and the associate learning subtest of the Wechsler Memory Test),^30^ and in the recognition of meaningless drawings.^26^ Anticholinergic medication may thus result in deficits in immediate recall of information, possibly reflecting its influence on processes of registration and short‐term memory deficits.^30^ Last, these changes were not modulated by dopaminergic therapy as l‐dopa withdrawal had no effect on tests of visual pattern and spatial recognition memory, visuospatial paired associative learning, and simultaneous and visual delayed‐matching‐to sample tasks.^6^, ^28^ Here, we complement these findings by showing a double dissociation between Ch1‐2, which correlated with memory and visuospatial tasks, and Ch3‐4, which correlated with global and fronto‐executive performances.

There are several limitations to this work. First, the basal forebrain is a small structure whose boundaries were difficult to delineate using MRI. Here we relied on previous histological studies of our team to determine the boundaries of the different nuclei of the basal forebrain.^32^ In addition, 2 raters performed segmentations independently with significant overlap. Reproducibility was better for Ch3‐4 (Dice = 0.79; Jaccard = 0.64) than Ch1‐2, which was more difficult to delineate (Dice = 0.64; Jaccard = 0.47). Regions that were difficult to distinguish were analyzed together (Ch1 and Ch2, Ch3 and Ch4). Last, to ensure better reproducibility of the results, we used a normalized mask of Ch1‐2 and Ch3‐4 that was coregistered to images of each participant. Second, fMRI and diffusion tensor imaging (DTI) detect changes in brain structure and connectivity, but the histological correlates of these changes are poorly understood and in particular they do not inform on the cholinergic nature of the neurodegenerative changes. Therefore, we cannot determine whether changes were only the result of degeneration of cholinergic neurons in the basal forebrain or to other types of neurons in this region as well. Third, the effects of dopaminergic medication on the functional changes observed here were not investigated.

## Conclusion

We found evidence of structural damage and connectivity deficits with target cortical regions in the basal forebrain of PD patients. Cognitive deficits associated with basal forebrain damage differed between Ch1‐2–hippocampal network (memory tests and the copy of Rey's figure) and Ch3‐4–cortical network (global cognition and executive functions). These results confirm that these 2 basal forebrain regions are affected in PD and further suggest that they contribute differently to the cognitive impairment in PD. Further longitudinal study will determine whether the extent of structural and functional alterations in the basal forebrain cholinergic nuclei is associated with increased risk of dementia in PD.

## Author Roles

1. Research project: A. Conception, B. Organization, C. Execution; 2. Statistical Analysis: A. Design, B. Execution, C. Review and Critique; 3. Manuscript Preparation: A. Writing of the first draft, B. Review and Critique

F.G.: 1A, 1C, 2A, 2B, 3A.

C.G.: 1C, 2C, 3B.

M.M.: 1C, 2C, 3B.

N.P.: 1B, 2C, 3B.

R.V.: 1C, 2B, 3B.

M.S.: 1A, 2C, 3B.

L.Y.‐C.: 2B, 3B.

M.V.: 1A, 2C, 3B.

S.L.: 1A, 1B, 2C, 3A, 3B.

## Financial Disclosures of all authors (for the preceding 12 months)

M.S. received grants from French Health Ministry (Programme Hospitalier de Recherche Clinique [PHRC] 2010 Iambio3, PHRC 2013 Shatau), Institut Roche de Recherche Médecine Translationelle (Imabio), IdRS (Institut de Recherche Servier, Imatau), UTB Foundation, and Lejeune Foundation. M.V. received unrestricted support for research on Parkinson's disease from Mr Jean‐Jacques Legrand (Société Francaise d'Esthétique). S.L. received grants support from Biogen and Fondation Bettencourt Schueller. F.G., C.G., M.M., N.P., R.V., and L.Y.‐C. have nothing to report.

## Supporting information

Appendix S1: Supporting Information
