# Supplementary material for: Multimodal magnetic resonance imaging investigation of basal forebrain damage and cognitive deficits in Parkinson's disease
Source: Mov Disord. 2018 Dec 10;34(4):516–25. doi: 10.1002/mds.27561 (PMC6590238; doi:10.1002/mds.27561)
Supplement: Supplementary file 1 — Appendix S1: Supporting Information [file MDS-34-516-s001.docx]

**Supplementary material**

**Histological procedure for delimitation of basal forebrain regions**

The histological sections were obtained from the brains of four subjects with no history of neurological or psychiatric illness (mean age ± standard deviation = 88 ± 3 years) obtained at routine autopsy. Blocks of tissue containing the basal ganglia and the nucleus basalis were fixed in parformaldehyde and picric acid, frozen in powdered dry ice, and cut into serial coronal sections (40 µm thick) on a freezing microtome. Sections taken at 720 µm intervals were stained using ChAT immunohistochemistry with a polyclonal antiserum against human ChAT as described in ^32^. Cholinergic structures included the septal nucleus (Ch1), the vertical limb of the diagonal band (Ch2), the horizontal limb of the diagonal band (Ch3), and the nucleus basalis of Meynert (Ch4) (Supplementary Figure 1). Histological sections were used to define boundaries of Ch1-4 in the MRI scans.

**Supplementary Figure 1.** **Comparison of** **histology-based and MR-based segmentation of Ch1-Ch4**.
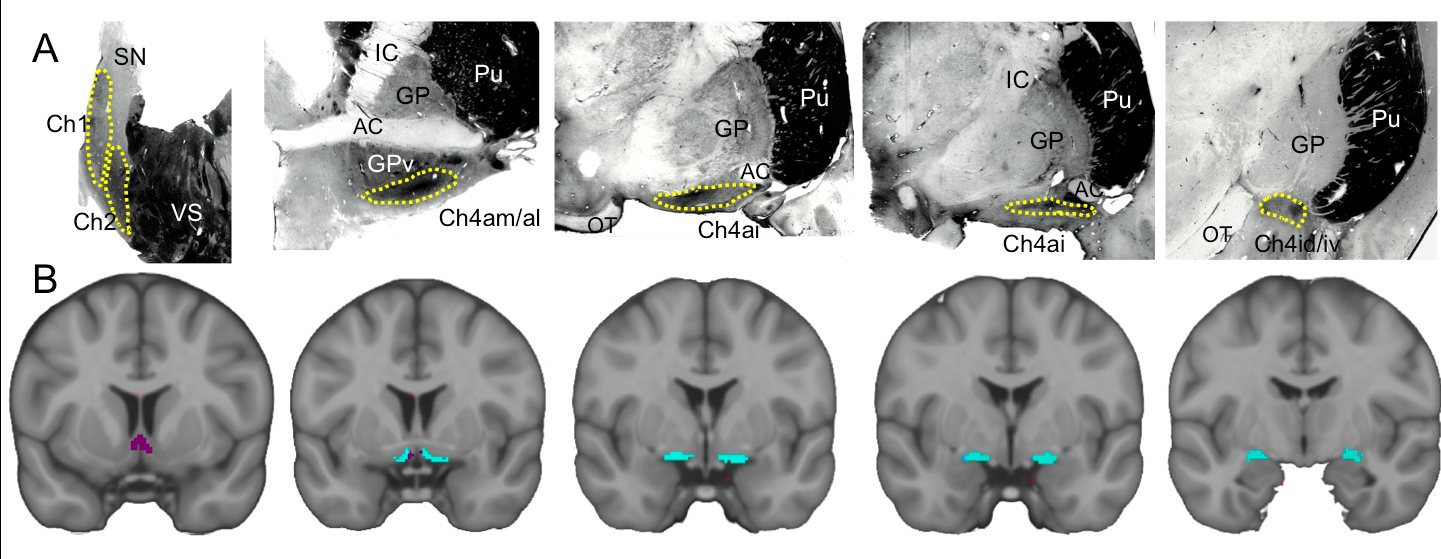


A) ChAT-stained coronal sections of the basal forebrain of one subject at the level of the septal nucleus (Ch1) and vertical limb of the diagonal band (Ch2), Ch3 and Ch4. Scale bar = 3 mm and applies to A and B. B) Coronal view of Ch1-2 (purple) and Ch3-4 (light blue) regions of interest superimposed on coronal T1-weighted MNI template image. Abbreviations. AC, anterior commissure, ChAT, choline acetyltransferase, Ch4am/al: anteromedial and anterolateral parts of Ch4, Ch4ai: anterointermediate part of Ch4, Ch4id/iv: intermediodorsal and intermediolateral parts of Ch4, GP: globus pallidus, GPv: ventral pallidum, IC, internal capsule, Pu: Putamen, SN: septal nuclei, OT: optic tract.

**Supplementary Figure 2.** **Probtrack streamlines from Ch3-4 to the cortex.**


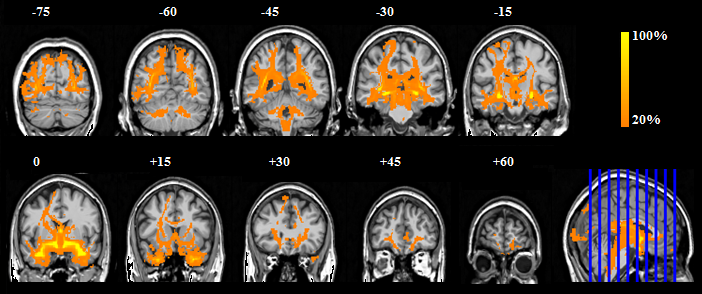


Mean FSL probtrack streamlines from bilateral Ch3-4 to the cortex in all control subjects. Individual maps were normalized by first coregistering diffusion and T1-weighted images and then applying the T1-weighted normalization parameters to the probtrack maps. The mean map is thresholded at 20% of the max number of fibers. As described by Mesulam et al. (2013) fibers were found in the white matter of the gyrus rectus and around the corpus callosum in the cingulate gyrus, as well as in the external capsule and uncinate fasciculus.^13^ Coordinates are in MNI space.

**Supplementary Figure 3.** **Maps of functional connectivity main effects for Ch1-2 and Ch3-4 in HV and PD patients.**


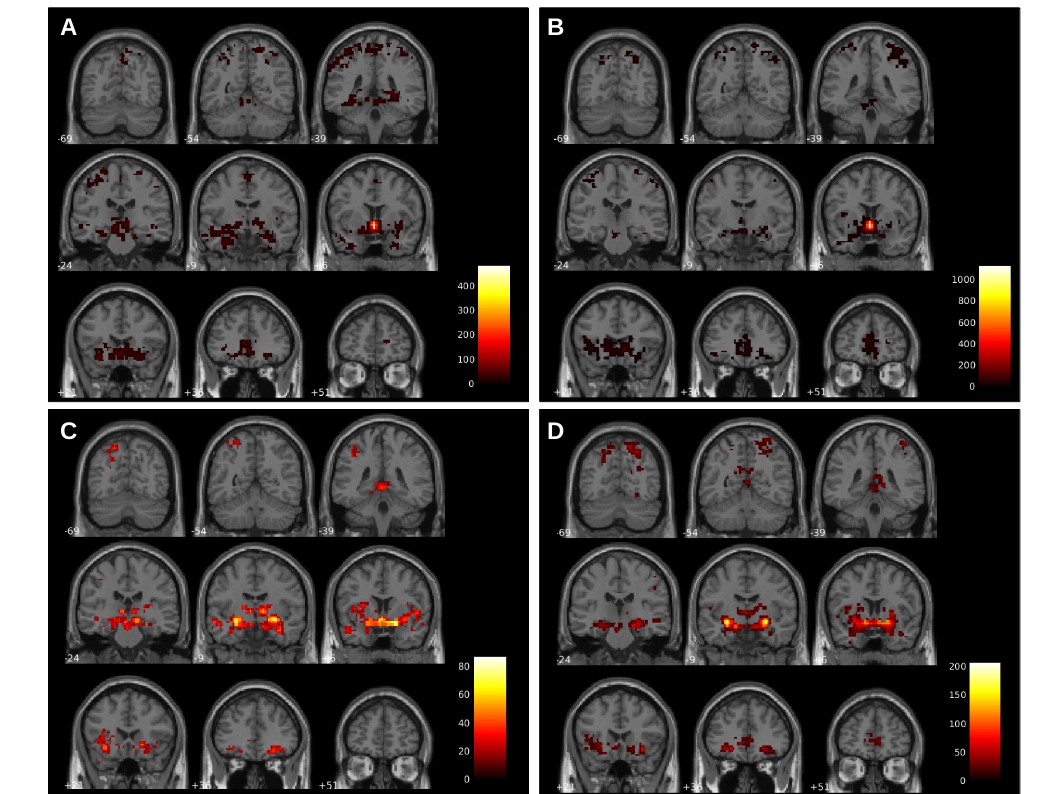


SPM group maps of functional connectivity main effects in the coronal plane of Ch1-2 (A and B) and Ch3-4 seeds (C and D) in healthy volunteers (A and C) and patients (B and D). Height threshold at p<0.001 uncorrected for multiple comparisons. Numbers indicate MNI coordinates. Color bar indicates z-scores levels.

**Supplementary Figure 4.** **Anatomical representation of the functional connectivity of Ch3-4 using a threshold of p<0.01 uncorrected for multiple comparisons.**
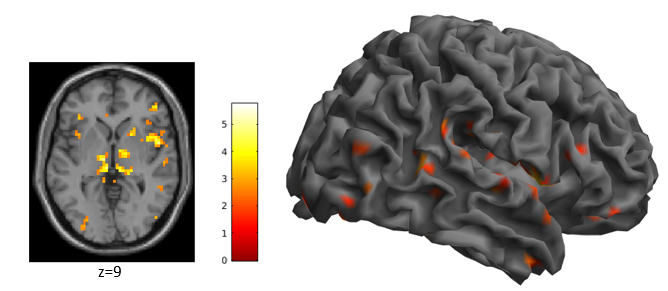


PD patients showed reduced functional connectivity between Ch3-4 and the temporal and the posterior regions. Clusters significant at p<0.01 uncorrected, left is left.

**Supplementary Figure 5. Correlations between MMSE scores and the probability of connection density of Ch3-4 and the prefrontal associative cortex**

30

28

26

24

22

0

5

10

15

x10^4^

Probability of connection density in the prefrontal Cortex

MMSE

r=0.42 (p<0.001)

Ch3-4

**Supplementary Figure 6. Exploratory analyses of correlations between RD and scores at the copy of Rey figure scores and TMT B-A test**.


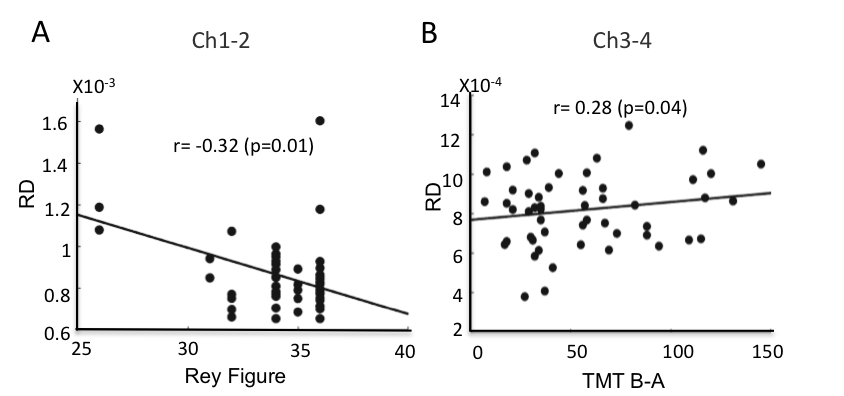


A) Significant negative correlations between scores at the copy of Rey Complex Figure and RD in Ch1-2. B) Significant positive correlations between scores at the TMT B-A and RD in Ch3-4.

**Supplementary Figure 7. Three-dimensional surface render of the correlation analyses between clinical scores and functional connectivity**. A) Functional connectivity changes in Ch1-2 correlated with i) Immediate Cued Recall (ICR) in bilateral superior, inferior and superomedial frontal gyri, and cingulate gyrus, the left middle frontal gyrus, putamen and hippocampus (red); ii) Total Free Recall (TFR) in the right superior frontal gyrus (yellow); and iii) Total Recall (TR) in the left inferior parietal lobe (green). Exploratory analyses also showed correlations with scores at the copy of Rey complex figure in the left middle occipital, superomedial frontal gyrus and calcarine area (blue). B) Functional connectivity changes in Ch3-4 correlated with scores at the MMSE in the right middle frontal gyrus, bilateral superior frontal gyri, the left middle cingulate gyri, and the right thalamus (red), and at the Stroop denomination test in the left supplementary motor area and lateral premotor cortex, bilateral postcentral and middle cingulate gyri, and the right superior parietal cortex (yellow).

**
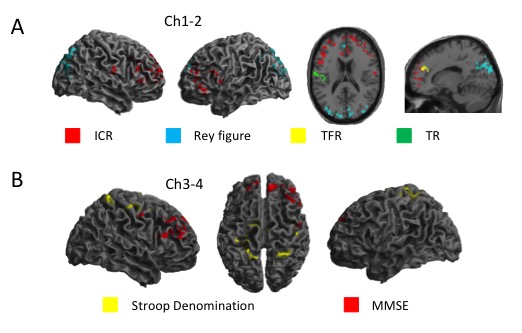
**

| \| **Supplementary Table 1. Density of connections of Ch1-2 and the hippocampus and Ch3-4 with the cortical and subcortical regions** \| \| \| \| \| --- \| --- \| --- \| --- \| \|  \|  \|  \|  \| \| **Mask** \| **Healthy volunteers** \| **PD patients** \| **ANCOVA p-value** \| \|  \| \| \|  \| \| **Ch1-2 – hippocampus** \| \| \|  \| \| Hippocampus \| 16.19 ± 7.67 \| 15.16 ± 3.31 \| 0.511 \| \|  \|  \|  \|  \| \| **Ch3-4 – cortical and subcortical regions** \| \| \| \| \| Associative Parietal \| 1935.51 ± 2411.92 \| 1138.04 ± 1488.99 \| 0.107 \| \| Associative Prefrontal \| 116.96 ± 286.61 \| 20.79 ± 28.48 \| **0.023** \| \| Associative Temporal \| 2669.47 ± 2878.61 \| 1846.82 ± 1738.87 \| 0.065 \| \| Limbic \| 2270.49 ± 1704.65 \| 2311.32 ± 1413.99 \| 0.498 \| \| Occipital \| 853.87 ± 1247.79 \| 388.24 ± 485.25 \| **0.027** \| \| Sensorimotor \| 139.67 ± 239.42 \| 105.08 ± 125.61 \| 0.600 \| \| Sub-cortical \| 2158.63 ± 2055.27 \| 1847.33 ± 1262.19 \| 0.316 \| \| Peri-insular cortex \| 548.24 ± 959.86 \| 156.05 ± 166.69 \| **0.016** \|   Cortical and subcortical regions defined using Freesurfer software. Values x 10^3^. Significant differences are indicated in bold. |
| --- | --- | --- | --- | --- | --- | --- | --- | --- | --- | --- | --- | --- | --- | --- | --- | --- | --- | --- | --- | --- | --- | --- | --- | --- | --- | --- | --- | --- | --- | --- | --- | --- | --- | --- | --- | --- | --- | --- | --- | --- | --- | --- | --- | --- | --- | --- | --- | --- | --- | --- | --- | --- | --- | --- | --- | --- | --- | --- | --- | --- | --- | --- | --- | --- |

| **Supplementary Table 2. Coordinates of regions showing reduced functional connectivity with Ch1-2 and Ch3-4 in PD** | | | | | | | |
| --- | --- | --- | --- | --- | --- | --- | --- |
| **ROI** | **Side** | **BA** | **MNI coordinates** | | | **Cluster size** | **T-score** |
|  |  |  | **x** | **y** | **z** |  |  |
|  |  |  |  |  |  |  |  |
| **Ch1-2** |  |  |  |  |  |  |  |
| Hippocampus | R |  | 27 | -36 | 3 | 50 | 5.46 |
|  | L |  | -21 | -33 | -6 | 31 | 4.05 |
| Parahippocampal gyrus | R |  | 30 | -42 | -6 | 50 | 4.28 |
|  | L |  | -18 | -36 | -15 | 31 | 4.49 |
| Middle temporal gyrus | L |  | -42 | -9 | -15 | 33 | 4.97 |
| Superior temporal gyrus | L |  | -45 | 9 | -21 | 33 | 4.35 |
| Fusiform gyrus | L |  | -24 | -30 | -24 | 57 | 4.68 |
|  |  |  |  |  |  |  |  |
| **Ch3-4** |  |  |  |  |  |  |  |
| Inferior frontal area | R |  | 51 | 3 | 3 | 29 | 5.60 |
| Thalamus | R |  | 9 | -27 | 0 | 22 | 4.38 |
|  | L |  | -9 | -27 | 3 | 38 | 4.75 |
|  |  |  |  |  |  |  |  |
| BA is Brodmann area |  |  |  |  |  |  |  |

| **Supplementary Table 3. Correlations between diffusion measures in the basal forebrain and scores at neuropsychological tests** | | | | |
| --- | --- | --- | --- | --- |
|  |  |  |  |  |
| **Region** | **Test** | **Diffusion measures** | **Correlation ( r )** | **P-value** |
|  |  |  |  |  |
| **Ch1-2** |  |  |  |  |
|  | | |  |  |
|  | Total Free Recall | |  |  |
|  |  | MD | -0,24 | 0.03 |
|  |  | RD | -0.24 | 0.03 |
|  | Copy of Rey figure | |  |  |
|  |  | MD | -0.35 | 0.02 |
|  |  | AD | -0.30 | 0.01 |
|  |  | RD | -0.30 | 0.02 |
| **Ch3-4** |  |  |  |  |
|  | | |  |  |
|  | Stroop denomination | | |  |
|  |  | MD | -0.28 | 0.01 |
|  |  | AD | -0.26 | 0.02 |
|  |  | RD | -0.28 | 0.01 |
|  | TMT B-A | |  |  |
|  |  | RD | 0.25 | 0.03 |
| **Fornix** |  |  |  |  |
|  | | |  |  |
|  | Total Free Recall | |  |  |
|  |  | FA | 0.28 | 0.04 |
|  |  |  |  |  |
|  |  |  |  |  |

Abbreviations. AD: axial diffusivity, MD: mean diffusivity, RD: radial diffusivity, TMT B-A: trail making test B-A.

| **Supplementary Table 4. Correlations between basal forebrain functional connectivity and scores at neuropsychological tests** | | | | | | | | | | |
| --- | --- | --- | --- | --- | --- | --- | --- | --- | --- | --- |
|  |  |  |  |  |  |  |  |  |  |  |
|  | **Test** | **Region** | **Side** | **BA** | **MNI coordinates** | | | **T-score** | **Correlation (r)** | **P-value** |
|  |  |  |  |  | **x** | **y** | **z** |  |  |  |
| **Ch1-2** |  |  |  |  |  |  |  |  |  |  |
|  | **Immediate cued recall** | |  |  |  |  |  |  |  |  |
|  |  | Superior frontal gyrus | R | 10 | 18 | 60 | 18 | 5.48 | 0.52 | <0.001 |
|  |  |  | L | 10 | -21 | 48 | 21 | 5.08 | 0.47 | <0.001 |
|  |  | Middle frontal gyrus | L | 46 | -42 | 39 | 18 | 7.30 | 0.64 | <0.001 |
|  |  | Inferior frontal gyrus | R | 9 | 51 | 30 | 21 | 6.17 | 0.51 | <0.001 |
|  |  |  | L | 47 | -42 | 33 | -6 | 6.05 | 0.57 | <0.001 |
|  |  | Superomedial frontal gyrus | R | 10 | 9 | 57 | 0 | 5.66 | 0.48 | <0.001 |
|  |  |  | L | 10 | -3 | 60 | 15 | 5.41 | 0.51 | <0.001 |
|  |  | Anterior cingulate gyrus | R | 32 | 3 | 39 | 9 | 4.50 | 0.43 | 0.001 |
|  |  |  | L | 32 | -12 | 27 | 27 | 5.14 | 0.48 | <0.001 |
|  |  | Hippocampus | L |  | -27 | -21 | -18 | 5.39 | 0.51 | <0.001 |
|  |  | Putamen | L |  | -27 | 12 | 6 | 5.45 | 0.49 | <0.001 |
|  | **Total free recall** | |  |  |  |  |  |  |  |  |
|  |  | Superior frontal gyrus | R | 9 | 18 | 42 | 30 | 4.32 | 0.36 | 0.008 |
|  | **Total recall** | |  |  |  |  |  |  |  |  |
|  |  | Inferior parietal cortex | L | 40 | -48 | -15 | 15 | 4.75 | 0.43 | 0.001 |
|  | **Copy of Rey figure** | |  |  |  |  |  |  |  |  |
|  |  | Middle occipital gyrus | L | 18 | -30 | -93 | 12 | 6.70 | 0.53 | <0.001 |
|  |  | Superomedial frontal gyrus | L | 9 | -6 | 48 | 30 | 5.27 | 0.43 | 0.001 |
|  |  | Calcarine area | L | 17 | 3 | -90 | -3 | 4.64 | 0.41 | 0.003 |
| **Ch3-4** |  |  |  |  |  |  |  |  |  |  |
|  | **MMSE** | |  |  |  |  |  |  |  |  |
|  |  | Middle frontal gyrus | R | 10 | 30 | 51 | 27 | 6.08 | 0.52 | <0.001 |
|  |  | Superior frontal gyrus | R | 9 | 15 | 51 | 39 | 5.21 | 0.46 | <0.001 |
|  |  |  | L | 9 | -21 | 48 | 33 | 4.71 | 0.44 | 0.001 |
|  |  | Middle cingulate gyrus | L | 24 | 0 | -18 | 42 | 4.72 | 0.48 | <0.001 |
|  |  | Thalamus | R |  | 12 | -24 | 12 | 4.36 | 0.45 | <0.001 |
|  | **Stroop denomination** | |  |  |  |  |  |  |  |  |
|  |  | Supplementary motor area | L | 6 | -6 | -3 | 72 | 4.87 | 0,42 | 0.002 |
|  |  | Lateral premotor cortex | L | 6 | -18 | -21 | 63 | 5.33 | 0.39 | 0.004 |
|  |  | Postcentral gyrus | R | 1-3 | 51 | -21 | 54 | 4.69 | 0.40 | 0.004 |
|  |  |  | L |  | -39 | -30 | 54 | 5.28 | 0.41 | 0.002 |
|  |  | Superior parietal cortex | R | 7 | 27 | -48 | 69 | 5.13 | 0.41 | 0.002 |
|  |  | Middle cingulate gyrus | R | 24 | 6 | -15 | 51 | 5.25 | 0.39 | 0.004 |
|  |  |  | L | 24 | -9 | -21 | 42 | 4.81 | 0.39 | 0.004 |
